# Spatial metabolomics and transcriptomics reveal cell type-specific dynamics in the placenta of patients with late-onset preeclampsia

**DOI:** 10.3389/fcell.2025.1659880

**Published:** 2025-10-08

**Authors:** Xiaowei Wei, Qian Li, Chuanmei Qin, Weihong Zeng, Yuxu Liu, Jiawei Lu, Jiuming He, Cailian Chen, Xiaoqing Zhang, Yi Lin

**Affiliations:** ^1^ Department of Obstetrics and Gynecology, Shanghai Jiao Tong University Affiliated Sixth People’s Hospital, Shanghai Jiao Tong University School of Medicine, Shanghai, China; ^2^ Department of Obstetrics and Gynecology, The International Peace Maternity and Child Health Hospital, Shanghai Jiao Tong University School of Medicine, Shanghai, China; ^3^ Shanghai Luming Biological Technology Co., Ltd., Shanghai, China; ^4^ State Key Laboratory of Bioactive Substance and Function of Natural Medicines, Institute of Materia Medica, Chinese Academy of Medical Sciences and Peking Union Medical College, Beijing, China; ^5^ Key Laboratory of System Control and Information Processing, Department of Automation, Shanghai Jiao Tong University, Ministry of Education of China, Shanghai, China; ^6^ Department of Pharmacy, The International Peace Maternity and Child Health Hospital, School of Medicine, Shanghai Jiao Tong University, Shanghai, China

**Keywords:** preeclampsia, spatial transcriptomics, spatial metabolomics, placenta, trophoblast

## Abstract

**Introduction:**

The placenta is vital for fetal development, but its growth can become disordered in pregnancy complications, particularly at the maternal–fetal interface. Preeclampsia, a severe condition that arises after the 20th week of pregnancy, is characterized by hypertension and other complications, posing significant risks to both mother and fetus. Despite its importance, the underlying mechanisms of preeclampsia remain poorly understood. Unraveling these mechanisms is essential for improving outcomes and advancing treatment strategies.

**Objectives:**

This study aimed to explore the spatial heterogeneity of the placenta and investigate the pathogenesis of late-onset preeclampsia (LOPE).

**Methods:**

We employed spatial transcriptomics (ST) and spatial metabolomics (SM) to map trophoblasts, fibroblasts, and immune cells, and analyze their transcriptomic and metabolomic profiles. A “spot-match” method was developed to integrate ST and SM data, revealing cell type-specific gene and metabolite changes during trophoblast differentiation.

**Results:**

The preeclamptic placenta showed increased fibroblasts and VCT proportions but a reduced SCT proportion. Complex interactions among trophoblasts, fibroblasts, and macrophages were observed in LOPE patients. Major metabolic reprogramming, particularly in glycerophospholipid and sphingolipid metabolism, was identified, potentially influencing trophoblast differentiation.

**Conclusion:**

Our ST and SM data offer new insights into LOPE mechanisms, providing valuable information for its prevention and treatment.

## Graphical Abstract

**Figure:**
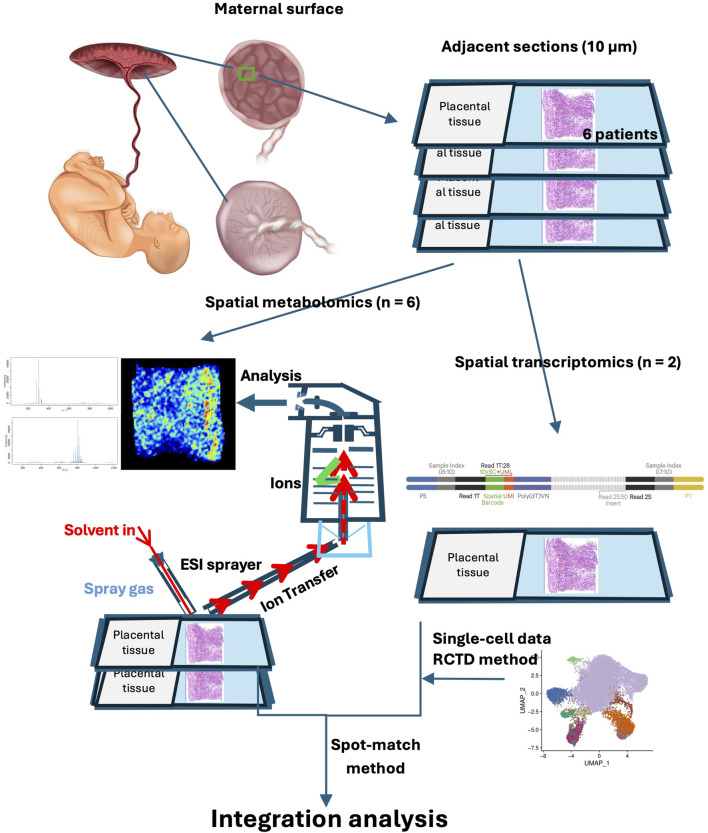
Spatial multi-omics reveal placental heterogeneity in late-onset preeclampsia.

## 1 Introduction

Preeclampsia is a serious pregnancy complication characterized by hypertension and additional symptoms, such as proteinuria and maternal organ dysfunction, occurring after 20 weeks of gestation. Affecting 2%–8% of pregnancies, it contributes significantly to maternal and fetal morbidity and mortality (Ives et al., 2020; Yang et al., 2021). According to the International Society for the Study of Hypertension in Pregnancy (ISSHP), preeclampsia is categorized as early-onset (EOPE, before 34 weeks) and late-onset (LOPE, at or after 34 weeks), reflecting different clinical and pathological mechanisms (Magee et al., 2022; Wójtowicz et al., 2019). Currently, the only definitive treatment is early delivery of the placenta, which often results in premature births. Understanding the molecular, immunological, and metabolic mechanisms of preeclampsia is critical for advancing prevention and treatment strategies.

While research has provided insights into EOPE, such as improving predictions through early screening and low-dose aspirin (De Borre et al., 2023; Chaemsaithong et al., 2022; Rolnik et al., 2022), the mechanisms underlying LOPE remain poorly understood. The classic two-stage hypothesis links EOPE to inadequate placentation and maternal systemic responses (Dimitriadis et al., 2023; Redman, 1991), but it inadequately explains LOPE, where neonates often reach term without growth restriction. Recent studies propose that LOPE may result from uteroplacental malperfusion at term, where placental compression disrupts intervillous perfusion and induces syncytiotrophoblast (SCT) stress (Weissgerber and Mudd, 2015; Staff, 2019). This “intrinsic” pathway highlights the need for detailed investigations into the spatial and metabolic mechanisms underlying LOPE.

Recent advances in single-cell sequencing, spatial transcriptomics (STs), and spatial metabolomics (SMs) provide powerful tools for addressing these gaps (Alexandrov et al., 2023). While single-cell RNA sequencing (scRNA-seq) excels at identifying cell types and gene expression at high resolution, crucial spatial information is lost because of the mechanical dissociation of tissue into single cells. This loss of spatial context obscures the true distribution and interactions of cells within their native environment. STs overcomes this limitation by providing gene expression profiles with near-single-cell resolution while preserving the spatial locations of cells within tissue sections (Williams et al., 2022). Similarly, SMs, through mass spectrometry imaging (MSI), enables the detailed mapping of metabolite distributions and concentrations within tissues at the micron scale (Santos et al., 2024; Li et al., 2024). The simultaneous application of STs and SMs provides a groundbreaking approach for studying the placenta in preeclampsia. This combined methodology uncovers the intricate spatial and functional architecture of the placenta, revealing the heterogeneous distribution of cell populations, variations in gene expression across different regions, and the spatial organization of metabolic processes. These findings would bridge the gap in spatial location in previous transcriptomic and metabolic studies of preeclampsia, thus advancing our knowledge of the mechanisms driving preeclampsia and identifying potential biomarkers and therapeutic targets.

In this research, we employed a multiomic strategy that integrates SMs and STs to develop a detailed spatiotemporal atlas of the placenta in LOPE. We analyzed the transcriptomic and metabolomic profiles of trophoblasts, fibroblasts, and immune cells in placental tissue and examined immune cell infiltration patterns and interactions with trophoblasts. Our study mapped the cell type-specific metabolic changes in the placenta, identifying a metabolic trajectory from villous cytotrophoblasts (VCTs) to SCTs and EVTs. Notably, we observed substantial metabolic reprogramming, particularly in glycerophospholipid metabolism, with STs revealing abnormal expression of key enzymes involved in this pathway, which drives the observed metabolic changes.

## 2 Materials and methods

### 2.1 Human placental tissue specimens

Participants were selected based on the diagnostic criteria for preeclampsia outlined in the 2019 ACOG Practice Bulletin, including individuals with late-onset preeclampsia (LOPE) and those with uncomplicated pregnancies (Author Anonymous, 2019). Women diagnosed with hypertension, kidney disease, cardiovascular disease, or other pregnancy complications (such as thyroid dysfunction, diabetes, and intrahepatic cholestasis of pregnancy) prior to conception were excluded. Additionally, women diagnosed with chorioamnionitis or with abnormal fetal chromosomes were excluded. Detailed information on these patients is shown in Supplementary Table S1. All patients underwent cesarean section to terminate the pregnancy. Immediately after placental delivery, fresh placental tissue near the maternal surface (adjacent to the decidua) was excised via sterile scissors. The tissue samples measured approximately 6.5 mm × 6.5 mm × 6.5 mm. For sampling, we avoided calcified areas, and the sites were located 2–5 cm from the center of the umbilical cord. The fresh tissue samples were blotted dry with paper towels to remove surface blood, rapidly placed in a histology plastic cassette and embedded in Cryo-Gel on dry ice. The samples were then stored at −80 °C for subsequent spatial transcriptomic and metabolomic analyses. All steps were completed within 15 min of placental delivery.

All procedures involving the participants were performed in compliance with relevant laws and institutional guidelines and have been approved by the Ethics Committee of the Medical Ethics Committee of the International Peace Maternity and Child Health Hospital of China Welfare Institute, Shanghai [Date: 2021, Approval number: (GKLW) 2021-17]. This study was conducted in accordance with the Declaration of Helsinki. All patients were fully informed, and the consent form of the spatial-omics study was obtained in written form. The privacy rights of human subjects have been observed.

### 2.2 Tissue processing

The frozen Cryo-Gel blocks were cut into consecutive sagittal slices approximately 10 μm thick, totaling approximately 10 slices, via a precooled cryostat microtome (Leica CM 1860 UV, United States) at −20 °C. One set of tissue sections was stained with H&E and imaged at 5x magnification via a Leica DM5500 B microscope (Leica Microsystems, Germany) to capture and merge images of the entire array. Two sets were thaw-mounted on positive charge desorption plates (Thermo Scientific, United States). The sections were stored at −80 °C before further Waters-DESI analysis. Another set was mounted onto Visium CytAssist Spatial Gene Expression slides for ST analysis. The remaining tissue slices were kept for backup.

### 2.3 Spatial transcriptomics

The tissue sections were processed following the manufacturer’s protocol for Visium CytAssist Spatial Gene Expression for Fresh Frozen (CG000614). First, the placental sections were fully immersed in chilled methanol, incubated upright at −20 °C for 30 min, and then stained with H&E. After staining, the sections were covered with a coverslip and imaged under brightfield conditions via the 3D HISTECH Pannoramic MIDI FL system at ×40 magnification. Once the coverslip was removed, a destaining step was carried out. Following destaining, the slides were processed as outlined in the user guide (Visium CytAssist Spatial Gene Expression, CG000495). Probe hybridization was performed at 50 °C for 16–24 h, followed by ligation at 37 °C for 1 h, and then incubation at 4 °C. The tissue removal enzyme and probe release mixture were combined and incubated at 37 °C for 30 min. The Visium CytAssist Spatial Gene Expression slide was then transferred to a new 6.5 mm Visium Cassette, where probe extension was conducted at 45 °C for 15 min. Finally, KOH mixture was added for elution, and the mixture was incubated at room temperature for 10 min.

### 2.4 Library construction and sequencing

A space ranger was used to capture the area of the tissue in the slide, which measured 6.5 mm × 6.5 mm. Each area contained 5,000 barcoded spots with a diameter of 55 μm, and the spot-to-spot distance was 100 μm. Library construction was performed via the Visium CytAssist Spatial Gene Expression kit for Fresh Frozen (PN-1000520 for Human, 6.5 mm). Post-library construction quality control was performed via an Agilent Bioanalyzer High Sensitivity chip. The average fragment size was determined from the Bioanalyzer trace. The expected average fragment size was 240 bp, which was used as the insert size for library quantification. The DNA libraries were subjected to high-throughput sequencing in paired-end 150-bp sequencing (PE-150) mode. The sequencing results were visually analyzed via Loupe Browser (10x Genomics).

### 2.5 ST data processing

The FASTQ files were aligned to the GRCh38 human reference genome with Space Ranger software (version 2.0.1) from 10x Genomics, and unique molecular identifier (UMI) counts were compiled for each barcode. The detection of tissue overlaying spots, as opposed to the background, was performed via image analysis. The filtered UMI count matrix was subsequently analyzed with the Seurat R package (version 4.3.0). Normalization of the data was conducted with Sctransform, which applies a regularized negative binomial model to detect genes with significant variability. For dimensionality reduction on the log‒transformed gene‒barcode matrices of highly variable genes, PCA was employed. The cells were clustered on the basis of marker genes, which were determined via graph-based techniques with the FindAllMarkers function (test.use = bimod) and visualized via a 2-dimensional UMAP algorithm via the RunUMAP function. DEGs were identified through the Seurat function FindMarkers (test.use = presto), applying thresholds of p < 0.05 and |log2foldchange| > 0.58 for significance.

### 2.6 Cell type identification and RCTD analysis

A public preeclampsia scRNA-seq dataset (available at https://github.com/JustMoveOnnn/preeclampsia/tree/main/single_cell_matrix/data) was utilized as a reference for integration with our ST profile via RCTD (version 1.1.0) (Yang et al., 2023). This approach addresses the challenge of the limited resolution of STs, which often results in multiple cell types being present within a single spot. By leveraging annotated scRNA-seq data, RCTD employs supervised learning to define and map cell-specific states within the spatial data, enabling the inference of cell-type composition and proportions at each spot. For implementation, the scRNA-seq data were preprocessed via the NormalizeData and ScaleData Seurat functions. Dimensionality reduction was achieved through PCA with the RunPCA function. Cell clustering was performed using graph-based methods with the FindClusters function, and visualization was carried out via 2D UMAP with the RunUMAP function. The marker genes for each cluster were determined via the FindAllMarkers function (test.use = presto). The creat.RCTD function was used with default parameters, requiring at least one cell per cell type and one UMI per pixel, while the run.RCTD function was configured with doublet_mode set to FALSE. The cell type for each spot was ultimately assigned according to the highest proportion principle.

### 2.7 Spatial metabolomics

The frozen slides prepared earlier were freeze-dried at −20 °C for 1 h followed by 2 h at room temperature prior to mass spectrometry imaging (MSI) analysis, which utilized a DESI source (Waters/Prosolia) coupled to a Q Exactive Orbitrap mass spectrometer (Thermo Fisher Scientific, USA). In negative mode, the solvent formula was composed of acetonitrile (ACN)/H2O (8:2) without formic acid, whereas in positive mode, the solvent formula was composed of ACN/H2O (8:2) containing 0.1% formic acid. The solvent flow rate was maintained at 1.5 μL/min, with a transporting gas flow rate of 45 L/min. The spray voltage was set to 7 kV, and the distance between the sample surface and the sprayer was 3 mm, which was also the distance from the sprayer to the ion transporting tube. Additionally, the MS resolution was set at 20,000, covering a mass range of 70–1,200 Da. The automated gain control (AGC) target was set to 2 × 10^6^, and the maximum injection time was 200 ms. The S-lens voltage was adjusted to 55 V, and the capillary temperature was maintained at 350 °C. The scanning speed of the x-axis (Vx) in the sample section was 0.2 mm/s with a 100 μm vertical step in the y direction.

### 2.8 SM data processing

Ion images were reconstructed after background subtraction via the Cardinal 3 software package. All MS images underwent total ion count (TIC) normalization at every pixel. Region-specific MS profiles were derived through meticulous alignment with high-spatial-resolution H&E images. Discrimination of endogenous molecules within distinct tissue microregions was accomplished via supervised statistical analysis, specifically orthogonal partial least squares discriminant analysis (OPLS-DA). Variable importance of projection (VIP) values derived from the OPLS-DA model were used to assess the relative contribution of each variable to group differentiation. The VIP value indicates the significance of each variable in classifying sample categories based on the first two principal components of the OPLS-DA model. Variables with VIP values greater than 1 were considered to have a substantial impact. Metabolites were identified as significant if their VIP values exceeded 1.0 and their corresponding p-values were less than 0.05. Furthermore, given the unique data structure obtained from MSI analysis, we applied t-SNE to reduce the dimensionality of the MS data at each pixel. Additionally, spatial shrunk centroid clustering (SSCC) was utilized for clustering MSI data, aiming to differentiate samples based on variations in ion abundance across pixels. The ions detected via Waters-DESI were annotated via the pySM 5 pipeline and an in-house SmetDB database (Lumingbio, Shanghai, China).

### 2.9 “Spot-match” method for ST and SM integration

Integrating STs with SMs to analyze intrinsic biological changes and identify key metabolic pathways presents several challenges. These challenges stem from differences in spatial information labeling, variations in experimental procedures for different tissue slices, and discrepancies in spatial distributions. Consequently, aligning the data spatially and achieving point‒to‒point matching between the two datasets is complex. To overcome these difficulties, we developed a novel method for point-to-point matching of ST and SM data. This method begins by selecting at least six evenly distributed spots across the sample range in STs, obtaining their corresponding barcodes, and marking these spots on the H&E‒stained image. The spatial information from the transcriptomic barcodes and metabolomics data is then converted into a unified coordinate system via specific formulas. For transcriptomics, the coordinates are calculated as UTX = (TransX/2 + 0.5) * transresolution and UTY = TransY * sqrt (0.75) * transresolution (Supplementary Table S2). For metabolomics, the coordinates are UMX = MetaX * metaresolution and UMY = MetaY * metaresolution (Supplementary Table S3). Next, the distance ratios between identified spots in both datasets are calculated to determine the scaling factor. This is done via the following formula: Ratio = sqrt [(UTXa-UTXb)2+(UTYa-UTYb)2]/sqrt [(UMXa-UMXb)2+(UMYa-UMYb)2] (Supplementary Table S4). The ratios are filtered to remove outliers, and the mean scaling factor is computed. Afterward, the rotation angles between corresponding spots in both datasets are assessed to check for sample rotation. The formula used is: Angle = arctan [(UMYa-UMYb)/(UMXa-UMXb)] -arctan [(UTYa-UTYb)/(UTXa- UTXb)] (Supplementary Table S5). If the deviation exceeds 5°, adjustments to the transformation are made. Finally, the conversion of SM coordinates to match those from ST is carried out using the final scaling factor, rotation angle, and central coordinates. This comprehensive method ensures accurate spatial alignment between ST and SM data, facilitating effective multiomic analysis.

### 2.10 Pseudotime analysis

Monocle was used to model the dynamic changes in cellular development based on key gene expression patterns. Initially, genes with significant variation in expression across cells were selected. These genes were then subjected to dimensionality reduction, and a minimum spanning tree (MST) was constructed to identify the longest path, representing the differentiation trajectories of cells with similar transcriptional features. Subsequent branch expression analysis was conducted to model gene expression associated with different branch fates. These branches were subsequently subjected to SMs to identify dynamic changes in metabolites during the differentiation trajectories of cells.

### 2.11 Cell communication analysis

CellChat was used to infer and analyze cell‒cell communication networks from scRNA-seq data. Significant ligand‒receptor interactions were identified through interaction probability calculations and perturbation tests. The overall cell‒cell communication network was constructed by aggregating the number and intensity of significant interactions between cell types. Additionally, CellPhoneDB was employed to assess the expression abundance of ligand‒receptor pairs. Receptors and ligands with expression levels exceeding 10% in specific cell types were considered, while pairs with p-values greater than 0.05 were excluded to ensure reliable evaluation of interactions.

### 2.12 Immunofluorescence

For frozen section fixation and immunofluorescence staining, the frozen section was first placed in a 37 °C oven for 10–20 min to dry and remove moisture. The slide was then immersed in a fixative solution for 30 min, followed by three washes in PBS on a decolorization shaker for 5 min each. Heat-mediated antigen retrieval was performed for 30 min, ensuring that excessive evaporation of the buffer was avoided, and the section did not dry out. After antigen retrieval, the slides were allowed to cool naturally and were washed three more times in PBS for 5 min each. Excess liquid was gently removed, a circle was drawn around the tissue using a histology pen, and 3% BSA was added to block non-specific binding, followed by incubation for 30 min. The primary antibody (HLA-G, 1:200, AB52455) was then added to the section, and the slide was placed flat in a humidified chamber and incubated at 4 °C overnight. After incubation, the slide was washed three times in PBS (pH 7.4) for 5 min each. The secondary antibody (CY3-labeled goat anti-mouse IgG, 1:300, GB21301) was added, and the slide was incubated in the dark at room temperature for 50 min. Following this, three additional washes in PBS were performed. DAPI staining was applied to counterstain nuclei, and the slide was incubated in the dark at room temperature for 10 min. Finally, autofluorescence was quenched using fluorescence quencher solution B for 5 min, followed by rinsing under running water for 10 min. The slide was mounted using an anti-fluorescence quenching mounting medium, and images were acquired with DAPI excitation at 330–380 nm (emission 420 nm) and CY3 excitation at 510–560 nm (emission 590 nm).

### 2.13 Statistical analysis

Statistical analyses were performed using R or GraphPad Prism software. Endogenous molecule discrimination across distinct tissue microregions was achieved through Orthogonal Partial Least Squares Discriminant Analysis (OPLS-DA). The Variable Importance in Projection (VIP) values from the OPLS-DA model were used to evaluate the contribution of each variable to group differentiation, with variables having VIP values >1 considered significant. Metabolites were deemed significant if their VIP values exceeded 1.0 and their p-values were <0.05. OPLS-DA models were further evaluated using standard diagnostics, including cumulative R^2^X, R^2^Y, and Q^2^ values, as well as permutation testing. For example, in the PE versus NC comparison, the model achieved R^2^X(cum) = 0.926, R^2^Y(cum) = 1.000, and Q^2^(cum) = 0.974, while permutation testing yielded negative Q^2^ values (Q^2^ = −0.235), confirming the robustness of the classification and excluding overfitting (Supplementary Table S8).

## 3 Results

### 3.1 Overview of the multiomic data of the placenta in the normal control (NC) and LOPE groups

**FIGURE 1:**
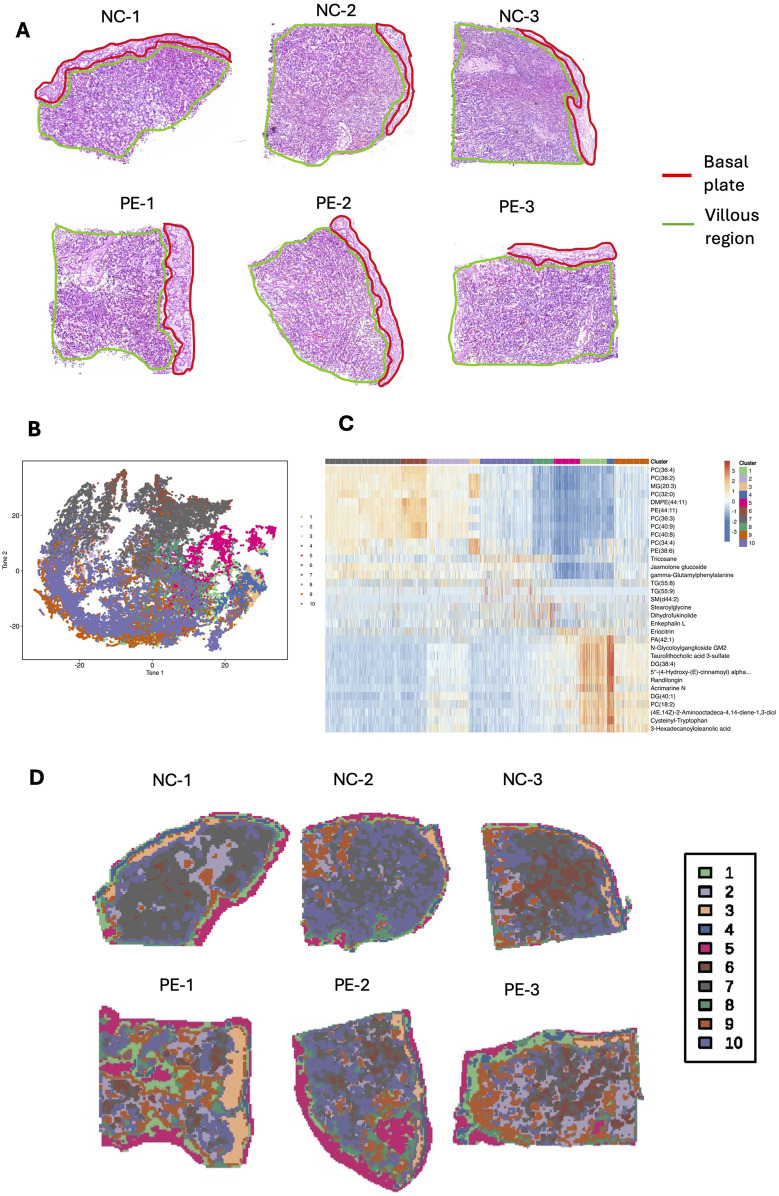
SM landscape of placental tissue from the preeclampsia and NC groups. **(A)** H&E staining image of placental tissue sections from three patients with preeclampsia and three NC individuals. **(B)** t-SNE was used for dimensionality reduction and visualization of the Waters-DESI data. **(C)** Heatmap showing the top 3 marker metabolites of each cluster. **(D)** SM feature plots of 10 clusters in six placental tissue samples.

To explore placental metabolic changes and interactions in preeclampsia, we developed a spatially resolved multiomic strategy to combine comprehensive molecular data from the maternal–fetal interface. The schematic workflow of sample collection and spatial multiomic detection is shown in the graphical figure. For spatial metabolomics, we analyzed three cases and three controls in total. Among these, one case and one control were also used for spatial transcriptomics. Placental tissue samples from six individuals were sectioned at 10 μm intervals. These samples were subjected to Waters-DESI for SM analysis, whereas two samples were additionally analyzed via the 10× Genomics Visium CytAssist system for ST analysis. This study employed a spatial resolution of 100 μm for integrating these diverse omics datasets. Figure 1A shows typical hematoxylin and eosin (H&E)-stained images of placental tissues, including the basal plate and villus region, from six patients (NC-1, NC-2, NC-3, PE-1, PE-2, and PE-3). Metabolite profiles were extracted from these images for SM analysis, and a data-driven segmentation map was created based on metabolite fingerprints. Using unsupervised t-distributed stochastic neighbor embedding (t-SNE) analysis, we identified and clustered the spatial expression patterns of tissue metabolites into ten primary clusters, which highlighted the main spatial features of the metabolite distribution (Figure 1B). The typical metabolites of each cluster are shown in a heatmap (Figure 1C), and the spatial shrunken centroid clustering (SSCC) of the placenta is shown in the image (Figure 1D). Cluster 1, 3, 4, and 5 were mainly distributed in the basal plate, while cluster 2, 6, 7, 9, and 10 were primarily demonstrated in the villous region. Cluster 8 was shown in both regions. These results suggest significant metabolomic heterogeneity in placental tissue. The differentially expressed metabolites and their spatial signatures were then analyzed and visualized. Principal component analysis (PCA) was initially used to evaluate the expression profiles of metabolites between the two groups, and the validated differentially expressed metabolites are shown in a heatmap (Supplementary Figures S1A,B). For the PE vs. NC comparison, the OPLS-DA model showed strong explanatory power (R2X(cum) = 0.926, R2Y(cum) = 1, R2 = 0.962) and excellent predictive ability (Q2(cum) = 0.974). Permutation testing yielded negative Q^2^ values (Q2 = −0.235), indicating that the classification was not driven by overfitting. Together, these diagnostics confirm that the OPLS-DA model is robust and reliable. Kyoto Encyclopedia of Genes and Genomes (KEGG) pathway analysis revealed that the differentially expressed metabolites were enriched in necroptosis, sphingolipid metabolism, and glycerophospholipid metabolism (Supplementary Figure S1C). A lollipop map showed that diacylglycerol (DG) (38:4) and N-glycoloylganglioside GM2 were significantly increased in the placenta of patients with preeclampsia, whereas phosphatidic acid (PA) (42:0) and phosphatidylcholine (PC) (36:4) were significantly decreased (Supplementary Figure S1D). The spatial metabolic image of GM2 demonstrated that GM2 increased mainly in the villous region of the placenta of patients with preeclampsia (Supplementary Figure S1E).

**FIGURE 2:**
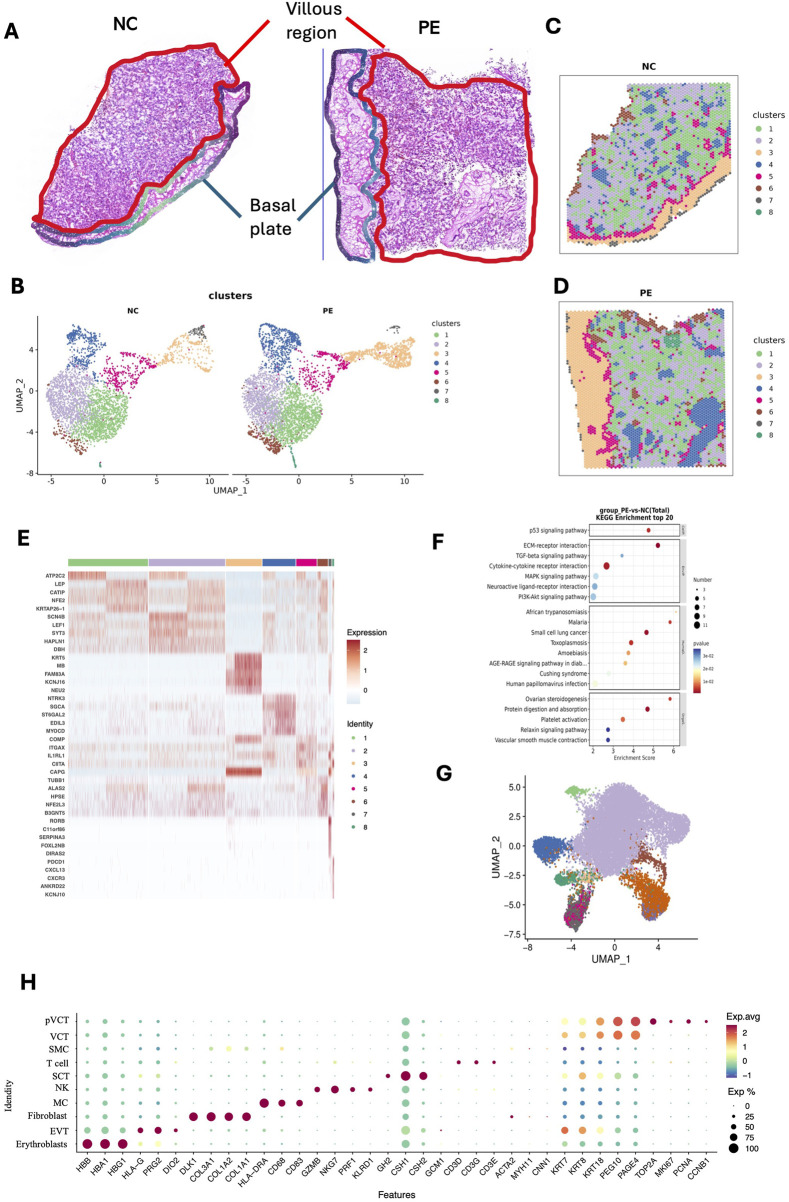
ST landscape of placental tissue from patients with preeclampsia and NC individuals. **(A)** H&E staining image of the placental tissue section from one patient with preeclampsia and one NC individual, demonstrating the villous region and the basal plate. **(B–D)** Unsupervised clustering analysis (UMAP) plots, and ST feature plots of 8 clusters in two placental samples. **(E)** Heatmap showing the top 5 marker genes of each cluster. **(F)** KEGG was used for differential gene enrichment analysis between patients with preeclampsia and NC individuals. **(G)** UMAP showing the definitions of 10 cell types in the placenta, including erythrocytes, EVTs, fibroblasts, macrophages, NK cells, SCTs, T cells, SMCs, VCTs, and pVCTs, based on public single-cell data. **(H)** Dotplot showing the representative marker genes of each placenta cell type according to the public single-cell dataset (available at https://github.com/JustMoveOnnn/preeclampsia/tree/main/single_cell_matrix/data).

We further investigated the *in situ* transcriptional profiles and their variability in placental tissues via ST analysis. Two frozen placental sections (Figure 2A), which were adjacent to those used for SMs, were analyzed via the 10× Genomics Visium CytAssist platform. Dimension reduction and clustering were performed on the basis of the gene expression data, allowing us to visualize the spatial distribution of differentially expressed genes (DEGs). We applied uniform manifold approximation and projection (UMAP) to assess the transcriptomic diversity in the placenta (Figure 2B). The entire placental section was categorized into eight distinct clusters based on gene expression profiles from 3,581 to 4,632 spots, with an average of 5,574 to 5,620 genes per spot (Figures 2C,D). At this stage, we performed an initial categorization of the mixed-cell spots, guided primarily by the representative marker genes enriched in each cluster. Owing to the intrinsic resolution of spatial transcriptomics (with multiple cells captured per spot), this approach allows the identification of region-associated cellular compositions rather than single-cell identities. Clusters 3 (highlighted in yellow, characterized by DEGs such as KRT5 (Maxwell et al., 2023), NEU2 (Koseki et al., 2012), and KCNJ16 (Guo et al., 2022), and SPRR2G (Wedenoja et al., 2020)) and 7 (highlighted in gray, characterized by DEGs such as RORB (Gong et al., 2021)) were predominantly identified in the basal plate, as annotated by the pathologists. In contrast, the other clusters were primarily localized within the villous regions (Figures 2C,D). We annotated the biological characteristics of each cluster via the top ten marker genes, which generally aligned with the expected morphological features of the clusters (Figure 2E; Supplementary Figure S2A). According to the gene expression pattern, cluster 1 (KRTAP26-1 (Miller et al., 2017), LEP (Hogg et al., 2013), and CATIP (Laufer et al., 2022) and cluster 2 (LEF1 (Jang et al., 2020), and SYT3 (Lim et al., 2020) are villus-related regions. Cluster 4 (MYOCD and ST6GAL2 (Yung et al., 2023)) is a stroma-related region. Cluster 5 (ITGAX (Vondra et al., 2023), CIITA (Scharer et al., 2015), and IL1RL1) and 8 (CXCR3 and CXCL13) are immune-related regions. Cluster 6 is mainly composed of the HBM (May et al., 2011), ALAS2, and SLC4A1 genes, indicating that it is a hemoglobin-related cluster. Next, the DEGs of the whole placental section were analyzed, and KEGG enrichment analysis revealed significant involvement in cytokine‒cytokine receptor interactions, the PI3K‒Akt signaling pathway, and extracellular matrix (ECM)‒receptor interactions (Figure 2F).

### 3.2 Spatially defined cell patterns in the placenta and cell–cell interaction patterns in preeclampsia

**FIGURE 3:**
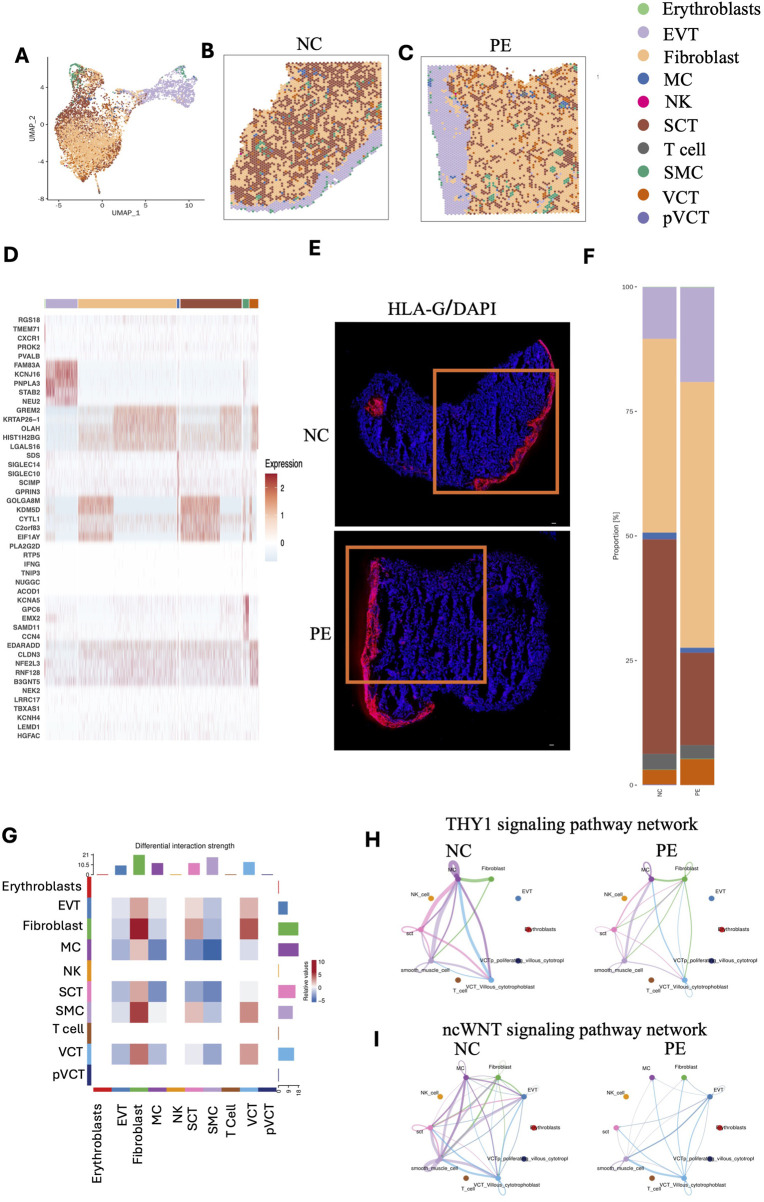
Mapping of cell types to spatial locations in two placental samples. **(A–C)** Mapping of cell types to spatial locations with RCTD in two placental samples. **(D)** Heatmap showing the top 5 differentially expressed genes for each cell type. **(E)** Immunofluorescence staining of HLA-G in placental tissue sections from patients with preeclampsia and NC individuals. Red fluorescence indicates HLA-G expression, while blue fluorescence indicates DAPI nuclear staining. **(F)** Bar chart showing the proportions of different cell types in placental tissue sections from patients with preeclampsia and NC individuals. **(G)** Interaction heatmaps showing the differential interaction strengths among different cell types, including erythrocytes, EVTs, fibroblasts, macrophages, NK cells, SCTs, T cells, SMCs, VCTs, and pVCTs. **(H,I)** Interaction plots showing the ncWNT signaling pathway network and THY1 signaling pathway network among different cell types. EVTs: extravillous trophoblasts; NK cells: natural killer cells; SCTs: syncytiotrophoblasts; SMCs: smooth muscle cells; VCTs: cytotrophoblasts; pVCTs: proliferating cytotrophoblasts.

Given the limited spatial resolution of STs (with an average of 2–30 cells per spot), we integrated public scRNA-seq of placentas in the LOPE group and performed RCTD analysis (see Methods) on each spot to calculate the proportion and distribution of cell types in placental STs (available at https://github.com/JustMoveOnnn/preeclampsia/tree/main/single_cell_matrix/data) (Yang et al., 2023). RCTD is a supervised learning approach that decomposes cell type mixtures on the basis of profiles learned from scRNA-seq data, correcting for differences across sequencing technologies. This method maps cell types spatially, revealing the spatial components of cellular identity and revealing new principles of cellular organization in tissues (Cable et al., 2022). First, we annotated cell types on the basis of public single-cell data (Figures 2G,H). ST data were deconvolved by 10 cell types from scRNA-seq with RCTD under multiple modes, including EVTs, SCTs, VCTs, proliferating VCTs, fibroblasts, T cells, natural killer (NK) cells, macrophages (MCs), smooth muscle cells (SMCs), and erythrocytes (Figures 3A–C).

For cell type assignment, if the highest proportion of a cell type at each spot exceeded 70%, it was designated as the cell type in this spot; otherwise, the second most prevalent cell type at each spot was selected. To visualize the cellular composition across spots, we additionally summarized the RCTD results in a pie chart (Supplementary Figure S2B), in which the dominant cell type within each spot was selected as its representative annotation. This provides a straightforward overview of the predominant cellular identity in each spatial location. EVTs were distributed mainly in the basal plate, whereas SCTs, VCTs, and fibroblasts were distributed in the villous region. The top 10 marker genes of each cell type are shown in the heatmap (Figure 3D). Further, we performed immunofluorescence staining on the same tissue sections using HLA-G, a well-known marker for EVTs (Figure 3E). The results from these experiments are fully consistent with the EVT distribution observed in our spatial transcriptomics data. Intuitively, the basal plate may be mistaken for decidua. It is important to clarify that the area where EVTs are predominantly distributed is referred to as the basal plate, which is part of the placenta sample (Borbely et al., 2014). The decidua basalis, on the other hand, remains attached to the uterine wall and is not included in the placenta specimen analyzed.

Compared with those in the NC group, the proportions of EVTs and VCTs in the placenta in the preeclampsia group were greater, whereas the proportion of SCTs was lower (Figure 3F). Each cell type performs distinct functions. Campbell et al. constructed a comprehensive deconvolution reference comprising 19 fetal and 8 maternal cell types derived from term placental villous tissue. By applying this reference to eight publicly available preeclampsia microarray datasets (173 controls and 157 cases), they demonstrated that preeclamptic placentas harbor an increased proportion of extravillous trophoblasts (EVTs). Their analysis further indicated that cell composition differences account for much of the transcriptomic divergence observed between preeclampsia and control placentas (Campbell et al., 2023). Consistent with these results, our spatial multi-omic profiling revealed disruptions in trophoblast composition and function in late-onset preeclampsia. Collectively, these findings underscore the central role of trophoblast imbalance, particularly an overrepresentation of EVTs, in driving disease pathogenesis. KEGG analysis revealed that SCTs are primarily involved in the Hippo signaling pathway, PI3K‒Akt signaling pathway, and ECM‒receptor interactions. EVTs are predominantly associated with cancer pathways. Fibroblasts contribute mainly to cytokine‒cytokine receptor interactions, PI3K‒Akt signaling pathway, and ECM‒receptor interactions. DEGs in MCs were enriched primarily in the Rap1 signaling pathway, the apelin signaling pathway, and focal adhesion. In VCTs, the DEGs were notably enriched in focal adhesion, the PI3K‒Akt signaling pathway, and complement and coagulation cascades (Supplementary Figures S3A–E). In line with our results, Mizutani et al. found that inhibition of YAP/TAZ–TEAD activity was shown to induce CTB differentiation into SCTs, demonstrating that SCT development is directly regulated by the Hippo signaling pathway (Mizutani et al., 2022). A prior review highlights that EVTs share invasive, migratory, and immune-evasive properties with cancer cells, reinforcing their close association with tumor-like signaling programs, which is consistent with our finding (Pang et al., 2022).

We analyzed cell‒cell communication to explore how cell types, including EVTs, SCTs, VCTs, fibroblasts, and key immune cells such as MCs, interact with each other at the maternal–fetal interface. For this analysis, we utilized the established CellChat tool (Jin et al., 2021), which evaluates the interaction strength between cell types by assessing the reciprocal expression of all possible interactions. As shown in Figure 3G, the color gradient indicates the relative strength of interactions, with red representing stronger interactions and blue indicating weaker ones. The cell types shown include Erythroblasts, EVT, Fibroblast, MC, NK, SCT, SMC, T cell, VCT, and pVCT. Notably, fibroblasts show a strong interaction with VCT and SCT. This highlights the important role of fibroblasts in regulating trophoblast differentiation and possibly their involvement in placental remodeling (Ohmaru-Nakanishi et al., 2018). NK cells, T cells, and pVCT show very weak interactions, likely due to their low abundance within the placenta. As shown in Figures 3H,I, the THY1 signaling and the non-canonical WNT (ncWNT) signaling pathway network were reduced in the preeclampsia group compared with the NC group. THY-1 (also known as CD90) is a cell surface glycoprotein involved in various cellular processes, including cell adhesion, migration, differentiation, proliferation, and apoptosis, and may also modulate immune responses and vascular development (Rege and Hagood, 2006; Valdivia et al., 2023). Specifically in this study, THY1 signaling between MCs and SCTs or VCTs in preeclamptic placenta was reduced, suggesting a potential impairment of immune responses (Figure 3H). The ncWNT signaling pathway in the placenta refers to a branch of the Wnt signaling pathways that operates independently of the canonical Wnt/β-catenin pathway. In the placenta, ncWNT signaling plays a pivotal role in the development and function of trophoblast cells, which are essential for implantation, placental growth, and fetal development. This signaling pathway influences key cellular processes such as migration, differentiation, and blood vessel remodeling, all of which are critical for proper placental development. Additionally, ncWNT signaling regulates cell-cell interactions and cytoskeletal remodeling, both of which are crucial for the formation of the syncytiotrophoblast layer and the maintenance of normal placental function. Dysregulation of ncWNT signaling has been implicated in various pregnancy-related disorders, including preeclampsia, where improper placental development and inadequate blood flow may result from altered signaling pathways (Dietrich et al., 2022; Ujita et al., 2018). We found a reduced interaction of ncWNT signaling among MC, Fibroblasts, VCT, and SCT in the preeclamptic placenta compared to NC (Figure 3I).

### 3.3 Spatial dynamics of gene and metabolite changes during trophoblast differentiation

**FIGURE 4:**
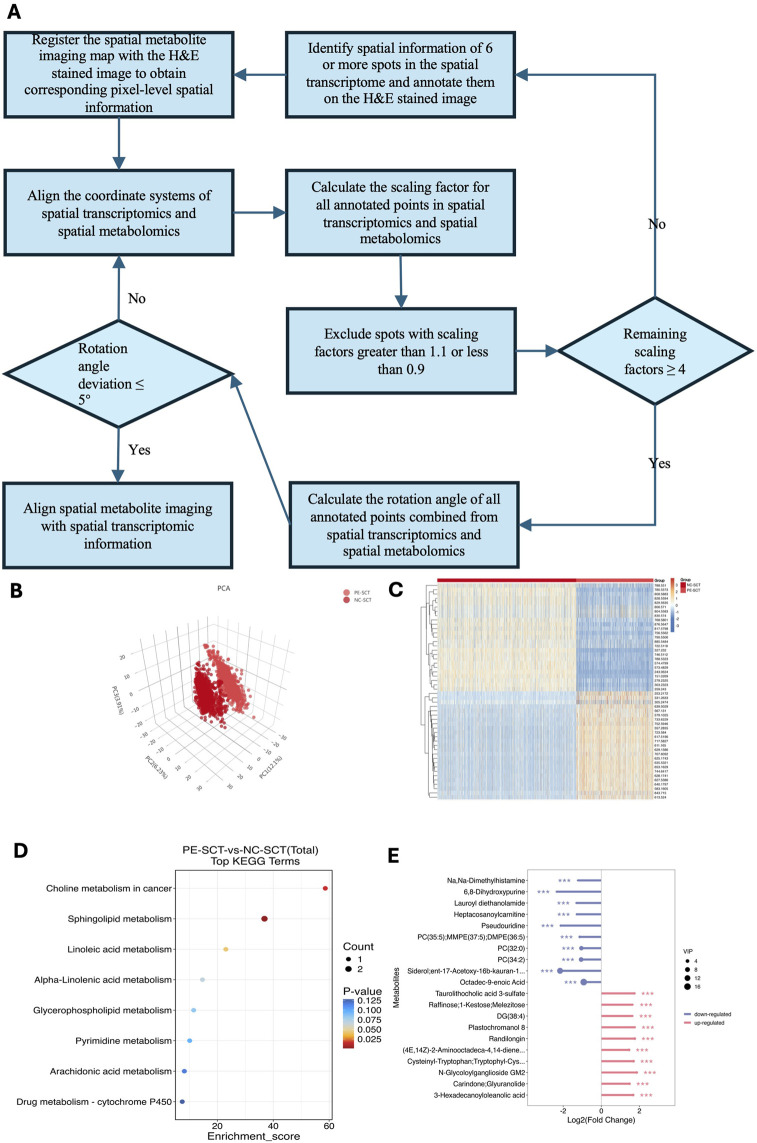
Differentially abundant metabolite ex pression in SCTs between patients with preeclampsia and NC individuals. **(A)** Schematic workflow of the “spot-match” method. This method begins by selecting at least six evenly distributed spots across the sample range in STs, obtaining their corresponding barcodes, and marking these spots on the H&E-stained image. The spatial information from transcriptomics barcodes and metabolomics data is then converted into a unified coordinate system via specific formulas. Next, the distance ratios between identified spots in both datasets are calculated to determine the scaling factor. The ratios are filtered to remove outliers, and the mean scaling factor is computed. Afterward, the rotation angles between corresponding spots in both datasets are assessed to check for sample rotation. If the deviation exceeds 5°, adjustments to the transformation are made. Finally, the conversion of SMs coordinates to match those from STs is carried out using the final scaling factor, rotation angle, and central coordinates. **(B)** PCA was used to reduce dimensionality and reveal key metabolite patterns in SCTs between patients with preeclampsia and NC individuals. **(C)** Heatmap showing the differentially abundant metabolites of the SCTs between patients with preeclampsia and NC individuals. **(D)** KEGG was used to identify and interpret biological pathways and networks that are significantly altered in SCTs. **(E)** Lollipop map was used to visualize the relative abundance of metabolites of SCTs between patients with preeclampsia and NC individuals.

At present, SM and ST analyses can only be conducted separately on adjacent placental sections. Despite the small distance of 10 μm between these sections, variations in section preparation and transfer can introduce spatial discrepancies. To better align the spatial features of SM and ST data from the placenta, we developed a spot-matching method based on the alignment principle described by Ravi et al. (2022), which enabled accurate spatial registration between transcriptomic and metabolomic datasets and served as the foundation for our integrative analysis. This technique synchronizes the spatial data from both analyses, ensuring accurate point-to-point correspondence. Three key parameters, including the final rotation angle, final scaling factor, and spatial coordinate of the transcriptomic center, are used to adjust and align the spatial coordinates of the metabolomic data with those of the transcriptomic data (Figure 4A). This method begins by selecting at least six evenly distributed spots across the sample range in STs, obtaining their corresponding barcodes, and marking these spots on the H&E‒stained image. The spatial information from the transcriptomic barcodes and metabolomics data is then converted into a unified coordinate system via specific formulas. For transcriptomics, the coordinates are calculated as UTX = (TransX/2 + 0.5) * transresolution and UTY = TransY * sqrt (0.75) * transresolution (Supplementary Table S2). For metabolomics, the coordinates are UMX = MetaX * metaresolution and UMY = MetaY * metaresolution (Supplementary Table S3). Next, the distance ratios between identified spots in both datasets are calculated to determine the scaling factor. This is done via the following formula: Ratio = sqrt [(UTXa-UTXb)2+(UTYa-UTYb)2]/sqrt [(UMXa-UMXb)2+(UMYa-UMYb)2] (Supplementary Table S4). The ratios are filtered to remove outliers, and the mean scaling factor is computed. Afterward, the rotation angles between corresponding spots in both datasets are assessed to check for sample rotation. The formula used is: Angle = arctan [(UMYa-UMYb)/(UMXa-UMXb)] -arctan [(UTYa-UTYb)/(UTXa- UTXb)] (Supplementary Table S5). This method increases the integration and analysis of spatial data from both techniques, allowing identification of cell type-specific metabolic changes related to preeclampsia. For identification of metabolic changes in the SCTs of the preeclampsia and NC groups, PCA was conducted, and heatmaps of differentially abundant metabolites are shown in Figures 4B,C. KEGG analysis demonstrated that the differentially abundant metabolites in SCTs were enriched mainly in sphingolipid metabolism, arachidonic acid metabolism, and glycerophospholipid metabolism (Figure 4D). PC (32:0) and PC (34:2) were significantly decreased in the SCTs of patients with preeclampsia, whereas DG (38:4) and N-glycoloylganglioside GM2 were significantly increased in the SCTs of patients with preeclampsia (Figure 4E).

**FIGURE 5:**
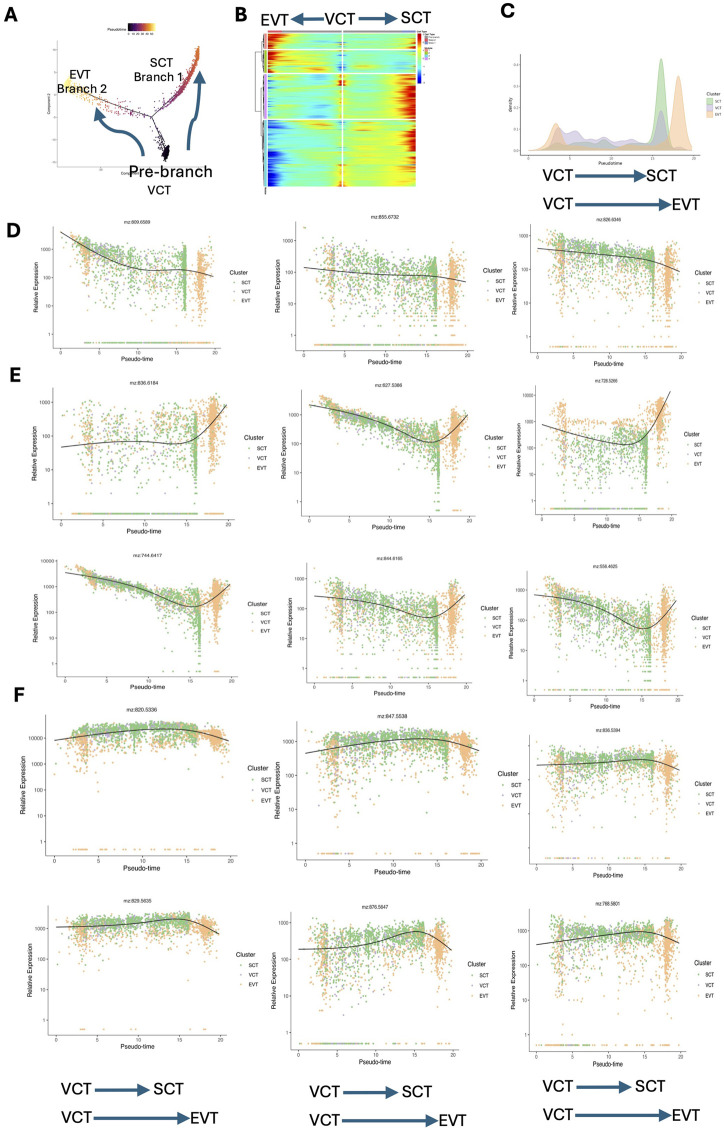
Pseudotime analysis revealing the dynamic metabolite and gene change patterns during trophoblast differentiation. **(A)** Trajectory reconstruction of trophoblast differentiation in placental tissues consisting of three branches: prebranch (VCTs), Branch 1 (SCTs), and Branch 2 (EVTs). Each point corresponds to a spot. **(B)** Heatmap plot displaying the expression patterns of pseudotime-specific genes associated with the trajectory of trophoblast differentiation. **(C)** Density plot illustrating the temporal changes in metabolic activity across VCTs, SCTs, and EVTs. This plot displays the frequency distribution of pseudotime scores assigned to each spot, with the pseudotime representing the progression of metabolic changes over time. **(D)** Sphingomyelin (m/z 809.6589, 855.6732), and PE (m/z 826.6346) decreased during the differentiation of VCTs to SCTs and EVTs. **(E)** PC (m/z 836.6184) remained unchanged during VCT to SCT differentiation but increased during VCT to EVT differentiation. DG (m/z 627.5386), PC (m/z 728.5266), GM2 (m/z 744.6417), LacCer (m/z 844.6165), and Cer (m/z 556.4625) decreased during VCT to SCT differentiation but increased during VCT to EVT differentiation. **(F)** PE (m/z 820.5336), PG (m/z 847.5538), PS (m/z 836.5394), phosphatidate (m/z 829.5653), PC (m/z 876.5647), and PC (m/z 768.5801) increased during VCT to SCT differentiation but decreased during VCT to EVT differentiation. PE: phosphatidyl ethanolamine; PC: phosphatidylcholine; GM2: GM2 ganglioside; LacCer: lactosylceramide; Cer: ceramide; PG: phosphatidylglycerol; PS: phosphatidylserine; EVTs: extravillous trophoblasts; SCTs: syncytiotrophoblasts; VCTs: cytotrophoblasts.

To explore the spatiotemporal transcriptomic and metabolic characteristics during the differentiation of trophoblasts, we selected spots in VCTs, EVTs and SCTs for pseudotime analysis via Monocle2. We constructed a trajectory of trophoblasts differentiation, in which the initiation of differentiation (Prebranch) occurred in the VCTs. The trajectory subsequently diverged into two branches (Branch 1 and Branch 2) on the basis of the difference in the transcriptional profile. SCTs were distributed mainly in Branch 1, whereas EVTs predominantly influenced the evolutionary directions of Branch 2 (Figure 5A). A Heatmap was used to display the expression patterns of pseudotime-specific genes in the trophoblast differentiation trajectory (Figure 5B). According to the results of point-to-point registration, the spot involved in VCT, EVT and SCT also has metabolite level data. Spots in different branches were then projected to spatial metabolism, and the trajectory of metabolic changes during trophoblast differentiation was depicted (Figure 5C). It is interesting to find a dual peak for EVTs in Figure 5C. This observation can be explained by the heterogeneity in EVT origins. While most EVTs are derived from the differentiation of VCTs, there is evidence suggesting that EVT subpopulations can arise directly from other sources, such as partially differentiated precursor cells or trophoblast stem cells (TSCs) in the early placenta. These TSCs have the potential to bypass the VCT stage and differentiate directly into EVTs or other trophoblast types, contributing to the dual-peak pattern observed (Nandi et al., 2018).

Sphingomyelin [d18:2(4E, 14Z)/24:2(5Z, 9Z)], Sphingomyelin (d42:0), and phosphatidyl ethanolamine (PE) (42:3) decreased during the differentiation of VCTs to SCTs and EVTs (Figure 5D). PC (38:2) remained unchanged during VCT to SCT differentiation but increased during VCT to EVT differentiation. DG (38:4), PC (32:3), GM2, lactosylceramide (LacCer) (d34:1), and ceramide (Cer) (d32:0) decreased during VCT to SCT differentiation but increased during VCT to EVT differentiation (Figure 5E). PE (44:11), phosphatidylglycerol (PG) (42:8), phosphatidylserine (PS) (40:6), phosphatidate (44:5), PC (20:1), and PC (18:0) increased during VCT to SCT differentiation but decreased during VCT to EVT differentiation (Figure 5F).

### 3.4 Dysregulated glycerophospholipid metabolism is a metabolic hallmark in preeclampsia

**FIGURE 6:**
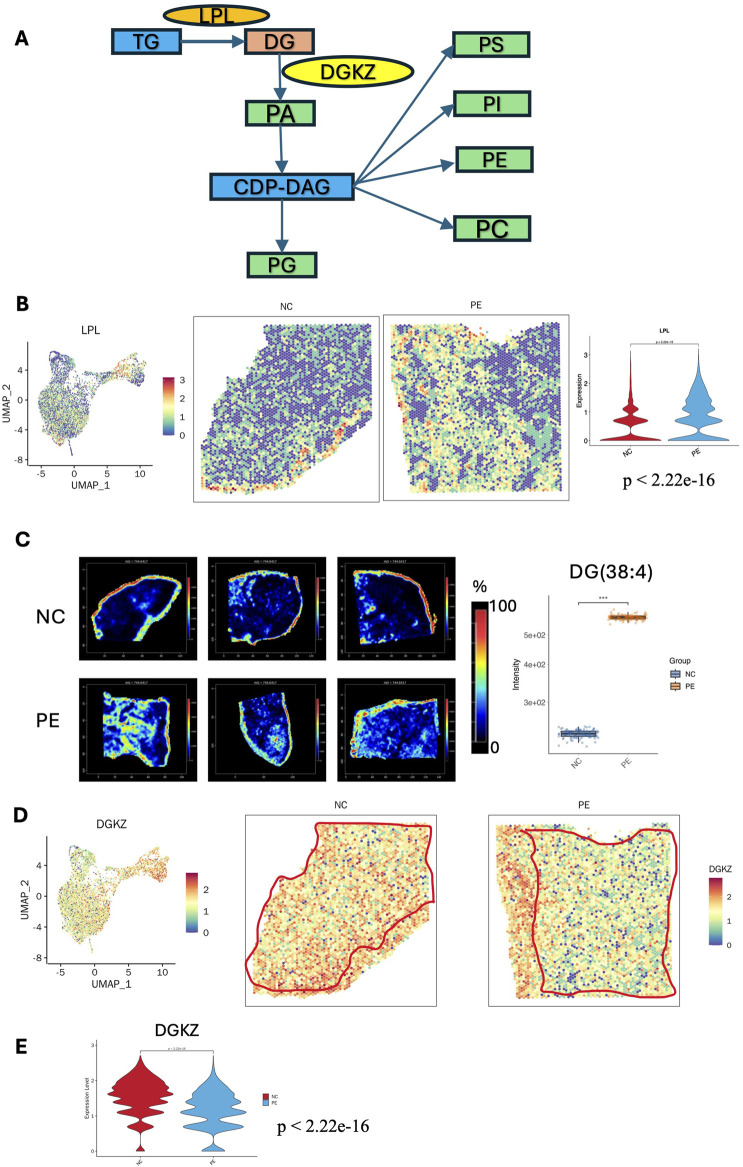
Metabolic reprogramming of glycerophospholipid metabolism in the placenta of patients with preeclampsia. **(A)** Schematic maps of glycerophospholipid metabolism: essential metabolites and enzymes involved in glycerophospholipid metabolism in the placenta of patients with preeclampsia. **(B)** STs images of the LPL gene in the placentas of patients with preeclampsia and NC individuals. Violin plot showing the LPL expression level in the villous region of the placenta was increased in the PE group compared with the NC group (p < 2.22e-16). **(C)** SM images of DG (38:4) in six placenta samples (intensity in the MS image color scale is the relative value, and the intensity in the gene image color scale is log2 transformed). The intensity of DG(38:4) was significantly increased in the PE group compared with the NC group (p < 0.001, statistical details are provided in Supplementary Table S6). **(D)** ST images of the DGKZ gene in the placentas of the preeclampsia and NC groups. **(E)** Violin plot showing the DGKZ expression level in the villous region of the placenta was decreased in the PE group compared with the NC group (p < 2.22e-16). LPL: lipoprotein lipase; DGKZ: diacylglycerol kinase zeta.

Phospholipids are essential components of cellular membranes and play crucial roles in signaling processes that are vital for cell growth and communication. Figure 6A shows a schematic of glycerophospholipid metabolism. Glycerophospholipids feature a glycerol backbone with three carbon atoms, and their synthesis involves modifications at the phosphate head group attached to the C3 position (Ziegler and Tavosanis, 2019). Lipoprotein lipase (LPL) plays a crucial role in converting triacylglycerol (TG) to DG, while the diacylglycerol kinase zeta (DGKZ) gene is essential for converting DG to PA. PC is the most prevalent phospholipid, while PE is the second most common phospholipid and is formed through headgroup exchange from PS (Butler et al., 2020). PS is synthesized through a headgroup exchange mechanism involving PC and PE. The key contributors to membrane lipid production include choline and ethanolamine (Joseph et al., 2020).

Analysis combining the ST and SM data revealed that, in the placental glycerophospholipid metabolic flux, products such as DG, PA, PG, PS, phosphatidylinositol (PI), PE, and PC, along with genes such as LPL and DGKZ, presented distinct distribution patterns. These findings indicate that disrupted glycerophospholipid metabolism is a key metabolic characteristic in the placenta and might critically contribute to the development of preeclampsia. Our results revealed that DG (38:4, m/z 627.53860) had a relatively high abundance in the SCTs of preeclampsia, while there was no significant difference in TG (46:6, m/z 803.55520) (Figure 6C). ST data revealed that the LPL gene was more abundant in the villous region of the preeclampsia group than in that of the NC group, which was consistent with the increased level of DG (38:4, m/z 627.53) (Figure 6B). DGKZ gene expression decreased in the villous region in preeclampsia, which was consistent with the distribution of its downstream metabolite PA (42:0, m/z 833.59400) (Figures 6D,E). Compared with that in the NC group, the level of PA (42:0, m/z 833.59400) in the SCTs and VCTs of the preeclampsia group decreased significantly (Supplementary Figure S4A). There was no significant difference in cyclic diphosphate diacylglycerol (CDP-DAG) (40:5, m/z = 1094.51880) in the placentas of the preeclampsia and NC groups. However, its downstream metabolites PG (36:2, m/z 775.54640), PE (20:3, m/z 788.53230), PC (32:0, m/z 756.55620), PS (38:3, m/z 794.53960), and PI (36:1, m/z 885.54840) showed a decreased distribution in the villous region of the preeclampsia group (Supplementary Figures S4B–D, S5A,B). Consistent with our KEGG analysis, a recent integrated metabolomic and lipidomic study of preeclamptic placentas demonstrated that both metabolites and lipids were predominantly altered in the glycerophospholipid metabolism pathway. Specifically, levels of PC, PE, and PS were decreased, whereas LPC and LPE were increased, reflecting abnormal membrane remodeling and activation of phospholipase A2–driven lipid turnover (Zhang et al., 2022). These disturbances are implicated in apoptosis, inflammation, mitochondrial stress, and ferroptosis, reinforcing the notion that glycerophospholipid dysregulation contributes critically to preeclampsia pathogenesis.

## 4 Discussion

We present the first study on preeclampsia addressing the clinically significant question of placental heterogeneity through spatial transcriptomics (ST) and spatial metabolomics (SM) techniques. Spatial omics is pivotal in understanding cellular heterogeneity and interactions within tissue microenvironments. While previous research extensively mapped placental transcriptomics, these studies lacked the spatial resolution needed to correlate gene expression with functional outcomes. Our integrative “spot-match” method combines ST, SM, and public single-cell sequencing data to identify distinct transcriptomic and metabolic patterns in LOPE placentas, uncovering cell type-specific metabolite profiles and mapping lipid metabolism pathways crucial to trophoblast differentiation.

The placenta is essential for pregnancy, mediating nutrient transfer, immune tolerance, and maternal–fetal adaptation (Pavličev et al., 2017). Its development involves critical processes such as the differentiation of trophoblast cells, the remodeling of maternal uterine vessels, and the establishment of immune tolerance at the maternal–fetal interface (Wei et al., 2022). The chorionic villus, the placenta’s functional unit, is composed of a stromal core, VCTs, and multinucleated SCTs, which facilitate the exchange of gases and nutrients between the mother and fetus. VCTs, which act as progenitor cells, either fuse to create SCTs or develop into EVTs that anchor the placenta and remodel uterine arteries (Liu et al., 2018). Disruptions in these placenta-specific cells, including VCTs, SCTs, EVTs, and macrophages are linked to placental diseases such as preeclampsia, where inadequate EVT invasion and uterine spiral artery dysfunction are major contributors (Pique-Regi et al., 2019). Our analysis revealed increased proportions of VCTs and fibroblasts but reduced SCTs in LOPE placentas compared to controls, reflecting impaired trophoblast differentiation. Our team previously demonstrated that dysregulated trophoblast syncytialization, mediated by MLL1 through epigenetic modulation of the Hippo signaling pathway, plays a key role in the development of preeclampsia (Wu et al., 2024).

Metabolic reprogramming was another key feature identified. Changes in lipid levels have been identified as potential risk factors for preeclampsia (Lu et al., 2021). Yang et al. suggested that abnormalities in lipid metabolism could be key risk factors for the onset and progression of preeclampsia (Yang et al., 2022). In this study, we found that sphingolipid metabolism and glycerophospholipid metabolism were significantly reprogrammed spatially in the placenta of patients with preeclampsia. We employed SMs to visualize the spatial distribution of various lipids, including DG, PC, PE, PS, PG, PI, and PA, within placental tissues. Our findings revealed several spatial patterns: most glycerophospholipids, except for DG, showed reduced levels in placental tissues affected by preeclampsia, whereas some sphingolipids, such as GM2, presented markedly increased levels in the villous regions of the placenta in patients with preeclampsia compared with those in the NC group. Concurrent ST analysis indicated that the expression of genes such as LPL and DGKZ, which are involved in phospholipid synthesis, also shifted accordingly. Similarly, He et al. also reported that PCs were generally decreased in preeclampsia, which is associated with alterations in phospholipid metabolism (He et al., 2021).

Sphingolipids, including Sphingomyelin, are vital for maintaining cell membrane stability and function (Hannun and Obeid, 2018). In preeclampsia, a condition characterized by oxidative stress, abnormal sphingolipid metabolism occurs. Studies have demonstrated that oxidative stress leads to excessive production of ceramides, which accumulate and increase autophagy in trophoblast cells (Melland-Smith et al., 2015). Gaudio et al. reported that Sphingomyelin levels are markedly elevated in the chorionic arteries of placentas affected by preeclampsia (Del Gaudio et al., 2020). Notably, lipid alterations strongly impact the differentiation of trophoblasts. Sphingomyelin (d42:0) and PE (42:3) decreased during the differentiation of VCTs to EVTs, whereas PC (38:2) increased during the differentiation to EVT. DG (38:4), PC (32:3), GM2, LacCer (d34:1), and Cer (d32:0) decreased during differentiation to SCTs, but increased during differentiation to EVTs. PE (44:11), PG (42:8), PS (40:6), PA (44:5), PC (20:1), and PC (18:0) increased during the differentiation of VCTs to SCTs, while decreased in the differentiation of VCTs to EVTs.

STs offers a major advantage by correlating RNA molecules to specific tissue areas, enabling the precise identification of molecular differences among various tissue and cell types. This capability is particularly valuable for distinguishing tissue types that lack clear histological boundaries, such as the villous regions in the placenta. A key limitation of STs is that each spot typically represents an average expression profile from multiple cells. In our study, we address this challenge by using RCTD to deconvolve these mixed profiles, allowing a more detailed identification of transcriptional programs within a broader tissue context. Building on the foundational work of Liu et al. (2022), Greenbaum et al. (2023), who explored STs at the maternal–fetal interface during early pregnancy, our research extends these findings by providing both metabolic and transcriptomic data from the placenta during late pregnancy. This advancement not only helps elucidate placental function and development over time but also offers new insights into the dynamic changes occurring at the maternal–fetal interface as pregnancy progresses.

A key innovation of this study was the development of the “spot-match” method, addressing alignment inaccuracies between ST and SM data caused by slice thickness variation. Previously, the integration of STs and SMs involved defining specific target regions on the ST and SM slices, calculating the average expression values for these regions, and then comparing them (Author Anonymous, 2019; Vicari et al., 2024; Chen et al., 2024). However, this method is limited by inaccuracies due to the variation in thickness between consecutive slices. To address this issue, we developed the “spot-match” method, which substantially improves accuracy by performing point-to-point integration of the ST and SM data. This approach calculates the final scaling factor, rotation angle, and central coordinates to ensure precise alignment.

This study has some limitations. The spatial metabolomics analysis was based on a relatively small number of samples (n = 3 per group), which, although acceptable for initial discovery in spatial omics studies, may reduce statistical power. The spatial transcriptomics cohort included only one sample per group and was not intended for between-group statistical comparisons. These limitations should be addressed in future studies through larger sample sizes and independent validation.

In the future, the integration of spatial proteomics is expected to shed light on key events in the development of preeclampsia. Combining these approaches will not only create a more detailed molecular map of the placenta at single-cell resolution but also help identify post-transcriptional modifications through advanced proteomics. Research should emphasize multiomic approaches to placental histopathology at the single-cell level. A broad range of samples from various placental subtypes will increase our understanding of the mechanisms underlying preeclampsia. Continued sharing of multimodal datasets will further contribute to our collective knowledge of complex placental disorders.

## 5 Conclusion

In conclusion, our study provides an exploratory demonstration of the integration of spatial metabolomics (SM) and spatial transcriptomics (ST) for characterizing metabolic remodeling and interactions within heterogeneous placental tissues. By jointly imaging and linking metabolite, lipid, and gene expression profiles, we highlight spatially convergent patterns and their associations with key metabolic pathways. These findings suggest trophoblast-associated metabolic dependencies and immune–metabolic alterations that may contribute to placental adaptation in late-onset preeclampsia. While limited by sample size, our results underscore consistent biological themes across modalities and offer hypothesis-generating insights that can guide future large-scale investigations and the development of potential therapeutic targets.

## Data Availability

The datasets used and/or analyzed during the current study are available from the corresponding authors on reasonable request.

## Supplementary material

SUPPLEMENTARY FIGURE S1 Differentially abundant metabolite analysis between patients with preeclampsia and NC individuals. **(A)** PCA was used to reduce dimensionality and reveal key patterns in the SM data. **(B)** Heatmap showing the differentially abundant metabolites of the placenta between patients with preeclampsia and NC individuals. **(C)** KEGG pathway analysis was used to identify and interpret biological pathways and networks that were significantly altered, providing insights into the functional impact of the observed metabolic changes. **(D)** Lollipop map was used to visualize the relative abundance of metabolites between patients with preeclampsia and NC individuals. **(E)** SM images of N-Glycoloylganglioside GM2 in six placenta samples. The intensity of GM2 was significantly increased in the PE group compared with the NC group (p < 0.001, statistical details are provided in Supplementary Table S6).

SUPPLEMENTARY FIGURE S2 Representative marker genes of each cluster and each cell type in the placenta. **(A)** Spatial expression images of representative genes in each cluster in the placental tissue sections. **(B)** Pie chart showing cell type compositions in each spot.

SUPPLEMENTARY FIGURE S3 KEGG analysis of different cell types between patients with preeclampsia and NC individuals. **(A)** KEGG analysis revealed that SCTs cells are primarily involved in the Hippo signaling pathway, PI3K‒Akt signaling pathway, and ECM‒receptor interaction. **(B)** Fibroblasts mainly function in cytokine‒cytokine receptor interaction, PI3K‒Akt signaling pathway, and ECM‒receptor interaction. **(C)** DEGs in MC cells are enriched primarily in the Rap1 signaling pathway, the apelin signaling pathway, and focal adhesion. **(D)** EVTs are predominantly associated with cancer pathways. **(E)** In VCTs, DEGs related to focal adhesion, the PI3K‒Akt signaling pathway, and the complement and coagulation cascades were notably enriched. KEGG: Kyoto Encyclopedia of Genes and Genomes; EVTs: extravillous trophoblasts; SCTs: syncytiotrophoblasts; VCTs: cytotrophoblasts.

SUPPLEMENTARY FIGURE S4 Visualization of the reprogrammed glycerophospholipid metabolic pathway in the placenta of patients with preeclampsia. **(A–D)** SM images of PA (42:0), PG (36:2), PC (32:0), and PE (20:3) in six placenta samples (intensity in the MS image color scale is the relative value, and the intensity in the gene image color scale is log2 transformed). The difference is statistically significant when p-value <0.05. Statistical details are provided in Supplementary Table S6.

SUPPLEMENTARY FIGURE S5 Visualization of the reprogrammed glycerophospholipid metabolic pathway in the placenta of patients with preeclampsia. **(A,B)** SM images of PS (38:3) and PI (36:1) in six placenta samples (intensity in the MS image color scale is the relative value, and the intensity in the gene image color scale is log2 transformed). The difference is statistically significant when p-value <0.05. Statistical details are provided in Supplementary Table S6.

SUPPLEMENTARY FIGURE S6
**(A,B)** UMAP revealing different cell types in the decidua and placenta based on public single-cell data. **(C,D)** The pie charts of cell proportions within each spatial spot in two placental samples. **(E,F)** Mapping of cell types to spatial locations with RCTD in two placental samples.

SUPPLEMENTARY TABLE S1 Clinical characteristics of placental tissue samples from patients with late-onset preeclampsia (PE) and normal controls (NC). Information includes maternal age, gestational age, parity, gestational age at PE diagnosis and delivery, fetal sex, sampling region (basal plate vs. chorionic villi), and distance from the cord insertion site.

SUPPLEMENTARY TABLE S2 Barcodes and spatial coordinates of representative spots in the spatial transcriptomics (ST) dataset. Each spot has a unique spatial barcode (Barcode), which can be used to identify its precise location on the tissue section. *TransX* and *TransY* denote the spatial coordinate values (x and y) assigned to each spot in the ST platform.

SUPPLEMENTARY TABLE S3 Spatial coordinates of representative spots in the spatial metabolomics (SM) dataset. *MetaX* and *MetaY* denote the x and y coordinate values assigned to each spot in the SM platform.

SUPPLEMENTARY TABLE S4 Scaling ratio results calculated from pairwise labeled spots. The table lists the Euclidean distances between paired locations in the spatial transcriptomics (ST) and spatial metabolomics (SM) datasets, together with the corresponding scaling ratios used to assess alignment accuracy.

SUPPLEMENTARY TABLE S5 Pairwise rotation angle values (in degrees) calculated between spatial transcriptomics (ST) and spatial metabolomics (SM) spots. These angles were used to evaluate rotational consistency across modalities in the alignment procedure.

SUPPLEMENTARY TABLE S6 List of differential metabolites identified between late-onset preeclampsia (PE) and normal control (NC) placentas. The table includes metabolite features with their annotation, m/z, compound class, and statistical parameters (VIP, fold change, p-value, FDR).

SUPPLEMENTARY TABLE S7 Representative list of lipid species identified in the analysis of characteristic metabolic alterations during the process of cellular development. *Full lipid IDs*correspond to annotated lipid molecules, with their measured m/z values and detected adduct types.

SUPPLEMENTARY TABLE S8 Summary of orthogonal partial least-squares discriminant analysis (OPLS-DA) model performance for PE vs. NC groups. The table reports model parameters including the number of predictive (PRE) and orthogonal (ORT) components, total components (N), cumulative explained variance (R2X(*cum*), R2Y(*cum*)), cumulative predictive ability (Q2(*cum*)), and cross-validation values (*R2, Q2*).

## Abbreviations

LOPE, late-onset preeclampsia; EOPE, early-onset preeclampsia; ST, spatial transcriptomics; SM, spatial metabolomics; scRNA-seq, single-cell RNA sequencing; MSI, mass spectrometry imaging; EVT, extravillous trophoblast; SCT, syncytiotrophoblast; VCTs, villous cytotrophoblasts; t-SNE, t-distributed stochastic neighbor embedding; SSCC, spatial shrunken centroid clustering; KEGG, Kyoto Encyclopedia of Genes and Genomes; DEGs, differentially expressed genes; RCTD, robust cell type decomposition; UMAP, uniform manifold approximation and projection; NK cells, natural killer cells; MCs, macrophages; SMCs, smooth muscle cells; TG, triacylglycerol; DGKZ, diacylglycerol kinase zeta; LPL, Lipoprotein lipase; PA, phosphatidic acid; DG, diacylglycerol; PC, phosphatidylcholine; PS, phosphatidylserine; PG, phosphatidylglycerol; LacCer, lactosylceramide; Cer, ceramide; PI, phosphatidylinositol; CDP-DAG, cyclic diphosphate diacylglycerol.
