# Supplementary figures and images for: Spatial metabolomics and transcriptomics reveal cell type-specific dynamics in the placenta of patients with late-onset preeclampsia
Source: Front Cell Dev Biol. 2025 Oct 8;13:1659880. doi: 10.3389/fcell.2025.1659880 (PMC12540468; doi:10.3389/fcell.2025.1659880)

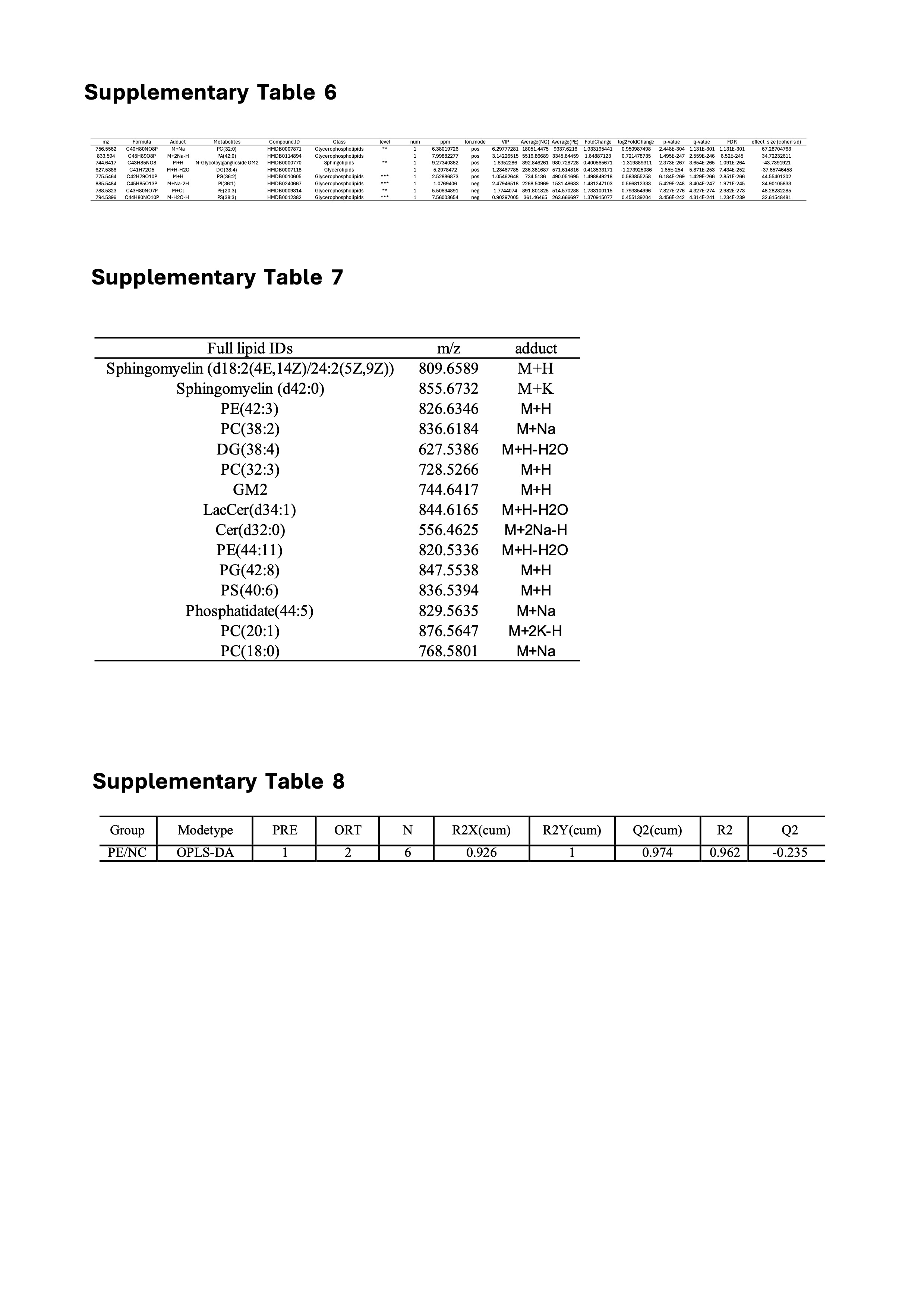

Supplement: Supplementary file 1 [file Image3.jpeg]

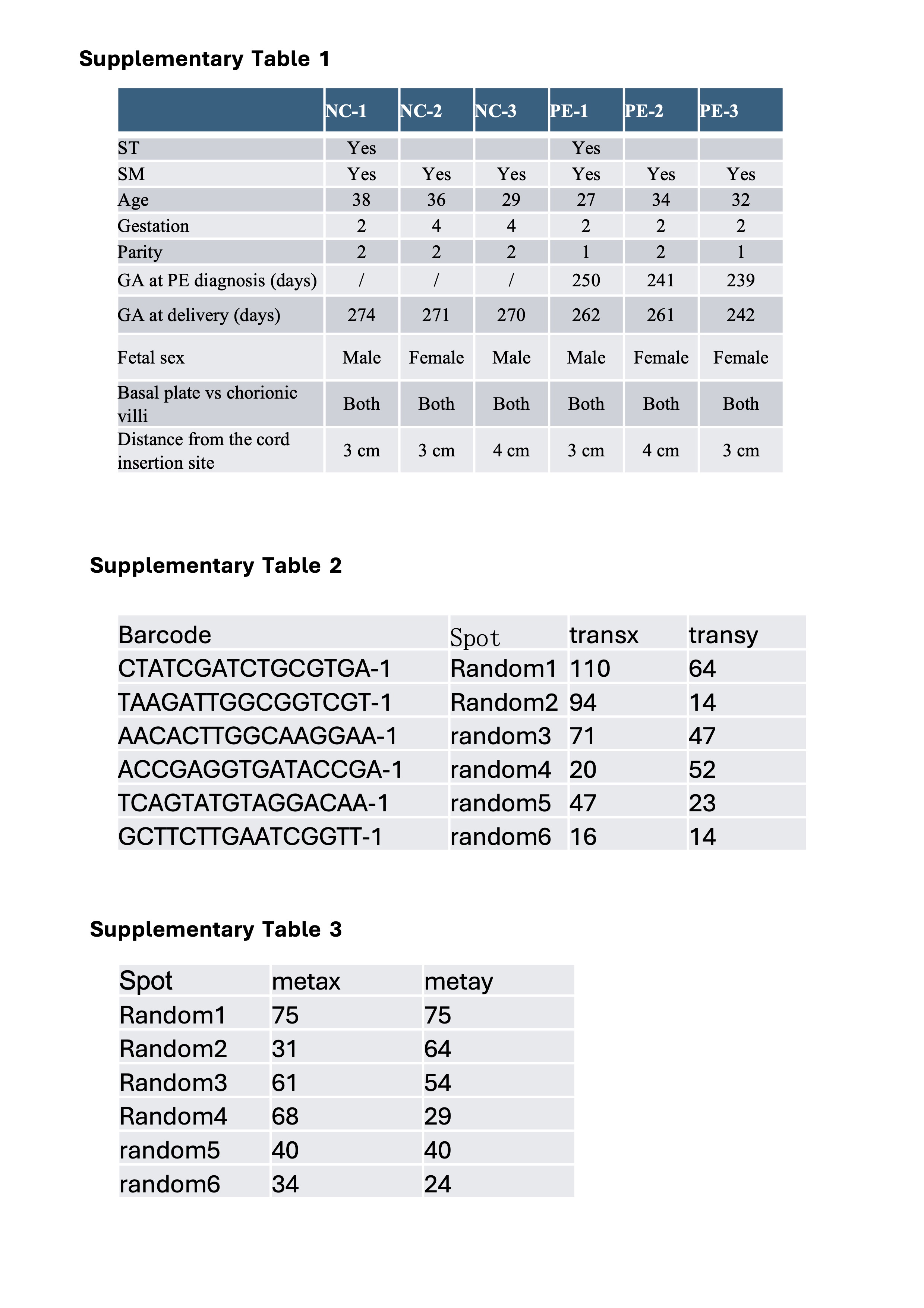

Supplement: Supplementary file 2 [file Image1.jpeg]

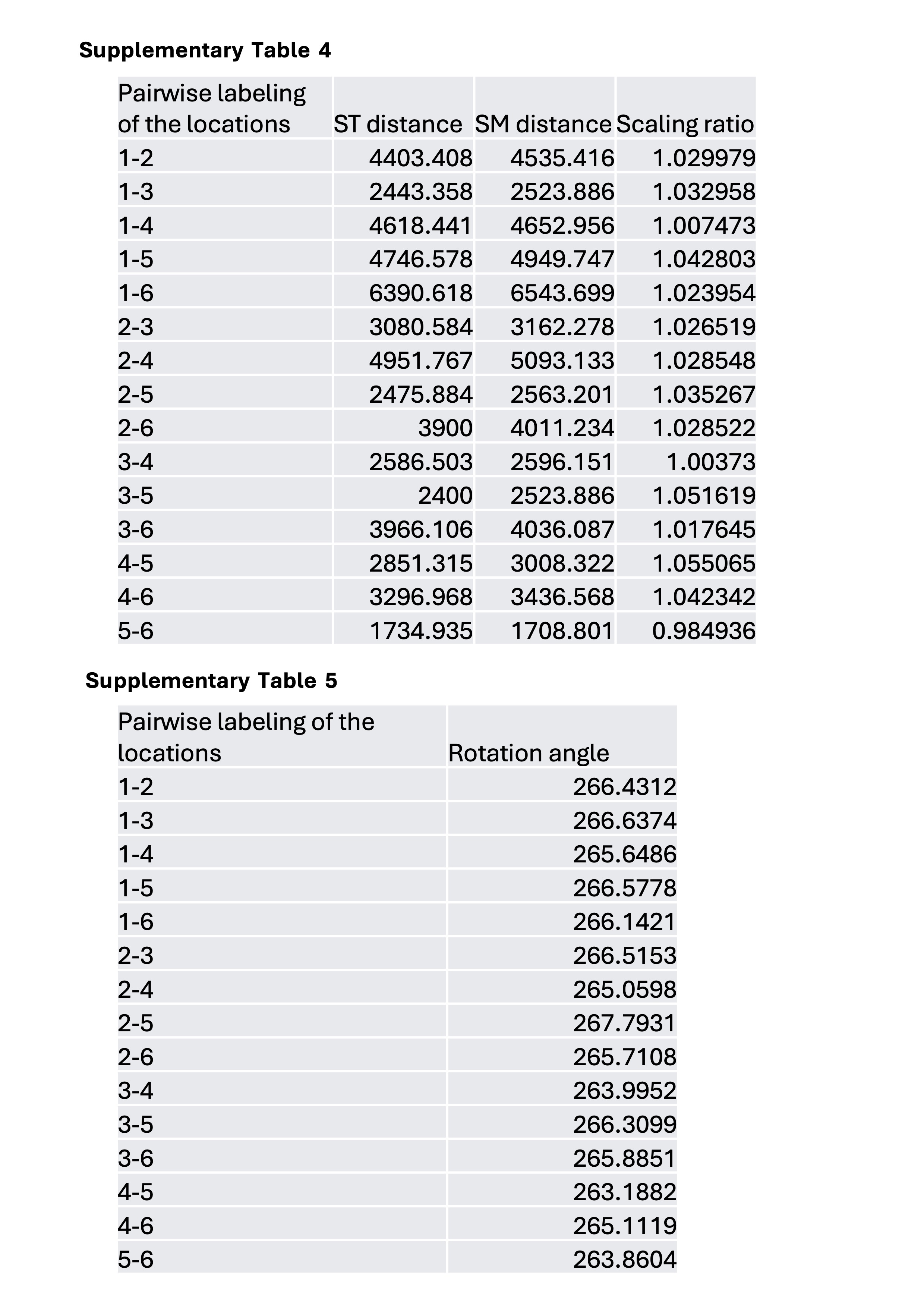

Supplement: Supplementary file 3 [file Image2.jpeg]
